# Signal averaging improves signal-to-noise in OCT images: But which approach works best, and when?

**DOI:** 10.1364/BOE.10.005755

**Published:** 2019-10-17

**Authors:** Bernhard Baumann, Conrad W. Merkle, Rainer A. Leitgeb, Marco Augustin, Andreas Wartak, Michael Pircher, Christoph K. Hitzenberger

**Affiliations:** Center for Medical Physics and Biomedical Engineering, Medical University of Vienna, Währinger Gürtel 18-20, 4L, 1090 Vienna, Austria

## Abstract

The high acquisition speed of state-of-the-art optical coherence tomography (OCT) enables massive signal-to-noise ratio (SNR) improvements by signal averaging. Here, we investigate the performance of two commonly used approaches for OCT signal averaging. We present the theoretical SNR performance of (a) computing the average of OCT magnitude data and (b) averaging the complex phasors, and substantiate our findings with simulations and experimentally acquired OCT data. We show that the achieved SNR performance strongly depends on both the SNR of the input signals and the number of averaged signals when the signal bias caused by the noise floor is not accounted for. Therefore we also explore the SNR for the two averaging approaches after correcting for the noise bias and, provided that the phases of the phasors are accurately aligned prior to averaging, then find that complex phasor averaging always leads to higher SNR than magnitude averaging.

## 1. Introduction

Optical coherence tomography (OCT) [1] performs biomedical imaging at high speed and with high sensitivity. State-of-the art OCT systems provide data rates of ∼100 million pixels per second and frame rates of ∼100-200 frames per second and beyond [2,3]. OCT provides a distinctively high sensitivity of typically ∼100 dB, meaning that backscatter signals as weak as $10^{-10}$ of a mirror reflection can still be detected [4–6]. At the same time, OCT enables imaging with a very high dynamic range spanning several tens of decibels between the strongest and the weakest signal in the image.

The strong performance of OCT in detecting weak signals can be improved even more by image processing. Noise in OCT images limits the detection capabilities for weakly scattering structures, and thus a variety of different approaches for reducing OCT image noise have been proposed [7–15]. The high acquisition speeds of modern OCT systems enable the improvement of the signal-to-noise ratio by averaging multiple signals, for instance by fusing OCT frames or even volumes quickly repeated at the same sample position. In recent years, several methods were proposed for improving the detection sensitivity of OCT by averaging the complex-valued OCT signals rather than just averaging their magnitudes [16–20]. In 2013, Szkulmowski and Wojtkowski published a thorough analysis of signals and noise subject to different averaging approaches [21]. In their analysis, the authors found a much stronger reduction of the noise floor by complex averaging as compared to magnitude averaging but also observed a heterogeneous outcome in terms of signal-to-noise performance for different imaging scenarios.

In this article, we set out to answer the question: Which signal averaging approach works best for improving signal-to-noise in OCT images, and when? We first introduce signals and noise in OCT (section 2.1) based on the analysis in Ref. [21]. We then analyze the signals and noise after magnitude and complex averaging, respectively, for a given pixel in an OCT image (section 2.2) and present simple expressions for the resulting signal-to-noise ratios. Next, we compare the somewhat surprising theoretical performance of the averaging schemes for different input signal levels and for different numbers of averaged signals and introduce a noise bias corrected signal-to-noise ratio (SNR) analysis (section 2.3). After substantiating our theoretical analysis with data from simulations (section 3.1) and experimentally acquired OCT data (section 3.2), we conclude the paper with a brief summary of the findings and their implications on actual OCT image processing (section 4). An overview of the terminology as well as a brief section on phase correction required for complex phasor averaging is provided in the appendix.

## 2. Analysis of signals and noise in OCT

### 2.1 OCT signals and noise

OCT signals $S_{OCT}$ can be described by complex phasors of the form

(1) $$S_{OCT}(x,t)=A(x,t)\exp\left[i\phi (x,t)\right]$$

where A(x,t) denotes the amplitude and ϕ(x,t) the phase of the OCT signal at location x and time t. Here the amplitude A(x,t) represents the length of the phasor and the phase ϕ(x,t) corresponds to the polar angle in the complex plane. The noise $S_{noise}$ in OCT images can be specified by phasors too, namely by random phasors. In the complex plane, the real and imaginary parts of random phasors, $r_{noise}$ and $i_{noise}$, can be described by normal distributions with zero mean and $\sigma^{2}$ variance [22]. Hence, the complex noise signal is characterized by a binormal (or Beckmann) distibution $p_{ri}$ in the complex plane [22,23]:

(2) $$p_{ri}(r_{noise},i_{noise})=\frac{1}{2\pi \sigma^{2}}\exp\left[-\frac{r_{noise}^{2}+i_{noise}^{2}}{2\sigma^{2}}\right].$$

This 2D Gaussian probability density function (PDF) describes the probability for a noise phasor of an image pixel to have the real part $r_{noise}$ and the imaginary part $i_{noise}$. The probability density function $p_{A}$ of the noise amplitude $A_{noise}$ is represented by the Rayleigh distribution (which can be derived by transforming Eq. (2) to polar coordinates and integrating over all angles ϕ) [22]

(3) $$p_{A}(A_{noise})=\frac{A_{noise}}{\sigma^{2}}\exp\left[-\frac{A_{noise}^{2}}{2\sigma^{2}}\right].$$

**Fig. 1.**
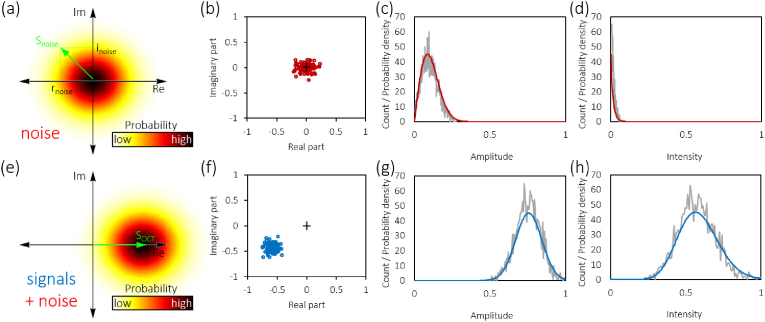
Complex phasor representation of noise and signals in OCT and their probability density functions. (a) Cartoon of Beckmann distribution of noise phasors around the origin of the complex plane. A representative phasor $S_{noise}$ with real part $r_{noise}$ and imaginary part $i_{noise}$ is shown in green. (b) Complex OCT signals of 100 repeated noise measurements in the same pixel from real-world OCT data. (c) Histogram of noise amplitudes (1000 repeats, gray line) and Rayleigh PDF (red line) computed from the standard deviation σ of the Beckmann distribution in (b) by Eq. (3). (d) Histogram of the noise intensity (gray line) and PDF (red line) computed from σ in (b) by Eq. (9). (e) Cartoon of an OCT signal affected by noise. The green arrow represents the signal phasor. (f) Complex OCT signals of 100 repeated measurements of a weak reflection in the same pixel. (g) Histogram of signal amplitudes (1000 repeats, gray line) and Rice distribution (blue line) computed from the mean signal amplitude and σ using Eq. (6). (h) Histogram of the signal intensity (gray line) and PDF (blue line) computed from the mean intensity and σ by Eq. (10). The + in (b) and (f) indicates the origin of the complex plane. The PDFs in (c,d,g,h) were scaled to match the count levels of the respective histograms.

Analogous to $p_{ri}$, the PDF of the noise amplitude represents the probability of the noise amplitude to take specific values $A_{noise}$. Figure 1(a-d)

shows an example of the complex representation of noise phasors in an OCT image and the corresponding Beckmann and Rayleigh distributions. Note that the Beckmann distribution is symmetric and centered at the origin while the Rayleigh distribution is skewed and only takes positive values. The mean amplitude $\bar{u}$ and variance $\sigma_{U}^{2}$ of a general PDF $p_{U}(u)$ is given by $\bar{u}=\int_{-\infty }^{\infty }up_{U}(u)du$ and $\sigma_{U}^{2}=\int_{-\infty }^{\infty }{\left(u-\bar{u}\right)}^{2}p_{U}(u)du$, respectively [22]. The mean amplitude $\bar{A_{noise}}$ and the variance $\sigma_{noise}^{2}$ of $p_{A}$ can be computed as

(4) $$\bar{A_{noise}}=\sqrt{\frac{\pi }{2}}\sigma ,$$

(5) $$\sigma_{noise}^{2}=\left(2-\frac{\pi }{2}\right)\sigma^{2}.$$

In every OCT image pixel, the detected signal is a combination of the actual OCT signal $S_{OCT}$ and the noise $S_{noise}$. By choosing the OCT signal to have ϕ=0 and thus to point into the direction of the positive real axis (Fig. 1(e)) similar to Goodman [22], the PDF of the measured amplitudes incorporating both signal and noise is represented by a Rice distribution

(6) $$p_{A}(A,A_{noise})=\frac{A_{noise}}{\sigma^{2}}\exp\left[-\frac{A_{noise}^{2}+A^{2}}{2\sigma^{2}}\right]{\text{B}}_{0}\left[\frac{A_{noise}A}{\sigma^{2}}\right].$$

where B_0_ is a modified Bessel function of the first kind and zero order. An example of the PDF of the signal amplitudes in presence of noise seen in an OCT image is shown in Fig. 1(g). Note that the signal gives rise to a somewhat spread probability accumulation at a high amplitude level. The mean amplitude $\bar{A}$ and variance $\sigma_{A}^{2}$ in an image pixel are observed as

(7) $$\bar{A}=\sqrt{\frac{\pi }{2}}\sigma L_{1/2}\left[-\frac{A^{2}}{2\sigma^{2}}\right],$$

(8) $$\sigma_{A}^{2}=A^{2}+2\sigma^{2}+\frac{\pi }{2}\sigma L_{1/2}^{2}\left[-\frac{A^{2}}{2\sigma^{2}}\right],$$

where $L_{1/2}(\cdot )$ denotes the Laguerre polynomial of degree 1/2. $L_{1/2}(-x)$ yields steadily increasing, positive values for increasingly negative arguments −x.

OCT amplitude data are usually squared in order to display a quantity proportional to sample reflectivity, $I=A^{2}$, henceforth called intensity. Therefore it makes sense to investigate the behavior of the PDFs of the squared signal and noise amplitude data. For this purpose, a variable transform of $A=\sqrt{I}$ is performed such that the PDFs are rendered into $p_{I}(I)=p_{A}(A=\sqrt{I})|\frac{dA}{dI}|$. The resulting PDFs for noise ($p_{I}(I_{noise})$) and signal intensities ($p_{I}(I,I_{noise})$) are

(9) $$p_{I}(I_{noise})=\frac{1}{2\sigma^{2}}\exp\;\left[-\frac{I_{noise}}{2\sigma^{2}}\right],$$

(10) $$p_{I}(I,I_{noise})=\frac{1}{2\sigma^{2}}\exp\;\left[-\frac{I_{noise}+I}{2\sigma^{2}}\right]\;{\text{B}}_{0}\;\left[\frac{\sqrt{I_{noise}I}}{\sigma^{2}}\right],$$

where $I_{noise}=A_{noise}^{2}$. Note that $p_{I}(I_{noise})$ is a negative exponential probability function whereas $p_{I}(I,I_{noise})$ is a combination of a negative exponential and a monotonically increasing Bessel function. Examples of the above PDFs for noise and signal intensities are shown in Figs. 1(d) and (h), respectively. The mean values and variances for noise and signal intensities, respectively, are given by

(11) $$\bar{I_{noise}}=2\sigma^{2},$$

(12) $$\sigma_{I_{noise}}^{2}=4\sigma^{4},$$

(13) $$\bar{I}=I+2\sigma^{2},$$

(14) $$\sigma_{I}^{2}=4\sigma^{2}(I+\sigma^{2}).$$

By comparing Eqs. (11) and (12), one can observe that the mean noise intensity equals the standard deviation $\sigma_{noise}$. Therefore, for the case of both non-zero signal and noise, the observed mean intensity corresponds to $\bar{I}=I+\bar{I_{noise}}$ and the intensity variance to $\sigma_{I}^{2}=\bar{I_{noise}}(2I+\bar{I_{noise}})$. Next, we will investigate the effect of averaging the magnitudes of the phasors as well as the effect of averaging the complex phasors on OCT intensity data.

### 2.2 Averaging magnitudes and phasors

For averaging OCT data, usually the absolute values (magnitudes) of the signals are used. For some applications, the complex signals have been exploited for adding or increasing image contrast in one way or another [31–36]. Here, we systematically analyze the effect of averaging magnitude and complex signals in OCT images.

#### 2.2.1 Magnitude averaging

Magnitude averaging uses the absolute values of N spatially and/or temporally separated signals (e.g., N pixels in a 2D or 3D kernel or N repeated measurements at the same spatial pixel location but at different time points):

(15) $$\langle I\rangle_{MAG}=\frac{1}{N}\sum_{j=1}^{N}I_{j}.$$

The PDF representing the average of N data points can be computed numerically by convolving the PDF of a single data point (*N* − 1)-times [21]. Unfortunately, no closed form expressions are available for the distribution describing an *N*-fold average of the above PDFs and only some approximations and bounds have been derived [37]. Nevertheless, the mean intensity values and intensity variances can be calculated for Rayleigh distributed noise and Rician signals affected by random noise, respectively, as

(16) $$\bar{\langle I_{noise}\rangle_{MAG}}=2\sigma^{2}=\bar{I_{noise}},$$

(17) $$\langle \sigma_{I_{noise}}^{2}\rangle_{MAG}=\frac{1}{N}4\sigma^{4}=\frac{1}{N}\sigma_{I_{noise}}^{2},$$

(18) $$\bar{\langle I\rangle_{MAG}}=I+2\sigma^{2}=\bar{I},$$

(19) $$\langle \sigma_{I}^{2}\rangle_{MAG}=\frac{1}{N}4\sigma^{2}(I+\sigma^{2})=\frac{1}{N}\sigma_{I}^{2}.$$

The above expressions show that the mean noise level as well as the observed mean signal level remain unchanged upon averaging and retain the mean intensity values calculated for single data points. However, the variances of the observed signal and noise, $\sigma_{I}^{2}$ and $\sigma_{I_{noise}}^{2}$, are reduced by 1/N and thus reducing the standard deviations $\sigma_{I}$ and $\sigma_{I_{noise}}$ by a factor $1/\sqrt{N}$.

#### 2.2.2 Complex averaging

In contrast to magnitude averaging, complex averaging also includes the phase information into the averaging process. This inclusion exploits the fact that the noise phasors (in absence of a signal) randomly fluctuate about the origin according to a 2D Gaussian PDF with a standard deviation of σ, see Eq. (2). By averaging N spatially and/or temporally independent complex noise phasors, the standard deviation σ of the Beckmann distribution in Eq. (2) is reduced by a factor $1/\sqrt{N}$ [22]. This reduction of the noise amplitude variance translates into a reduction of both the mean noise intensity as well as of its variance:

(20) $$\bar{\langle I_{noise}\rangle_{CPX}}=\frac{1}{N}2\sigma^{2}=\frac{1}{N}\bar{I_{noise}},$$

(21) $$\langle \sigma_{I_{noise}}^{2}\rangle_{CPX}=\frac{1}{N^{2}}4\sigma^{4}=\frac{1}{N^{2}}\sigma_{I_{noise}}^{2}.$$

Compared to the noise performance after magnitude averaging (Eqs. (16) and (17)), which maintains the mean noise level and reduces the noise intensity variance by 1/N, complex averaging leads to a 1/N decrease of the noise floor intensity and to a $1/N^{2}$ smaller noise intensity variance.

Recalling that we initially assumed that the signal vector $S_{OCT}$ was pointing in the direction of the positive real axis (i.e. Fig. 1(e)), the precondition for averaging signals in a complex fashion is that the phase of $S_{OCT}$ remains constant at ϕ=0. (Likewise, a different signal phase $\phi =\phi_{0}$ could be chosen, as long as the phases of all signals to be averaged are aligned at the same angle $\phi_{0}$. The signal phase (ϕ=0) is chosen here out of convenience to ensure a purely real signal which facilitates the calculations described above.) For such perfectly aligned signals in the presence of random noise, the following mean signal intensity and intensity variance will be observed:

(22) $$\bar{\langle I\rangle_{CPX}}=I+\frac{1}{N}2\sigma^{2}=\bar{I}-\frac{N-1}{N}2\sigma^{2},$$

(23) $$\langle \sigma_{I}^{2}\rangle_{CPX}=\frac{1}{N}4\sigma^{2}(I+\frac{1}{N}\sigma^{2})=\frac{1}{N}\sigma_{I}^{2}-\frac{N-1}{N^{2}}\sigma^{2}.$$

Unlike magnitude averaging, which maintains the original mean signal intensity $\bar{I}$, the mean signal intensity does not stay constant but is reduced by $2\sigma^{2}(N-1)/N$ after complex averaging. This signal reduction converges to an amount of $2\sigma^{2}$ for large N. At the same time, also the noise intensity variance is reduced by an amount $\sigma^{2}(N-1)/N^{2}$ compared to magnitude averaging, which converges to a reduction by $\sigma^{2}/N$ for large N.

### 2.3 Signal-to-noise performance

**Fig. 2.**
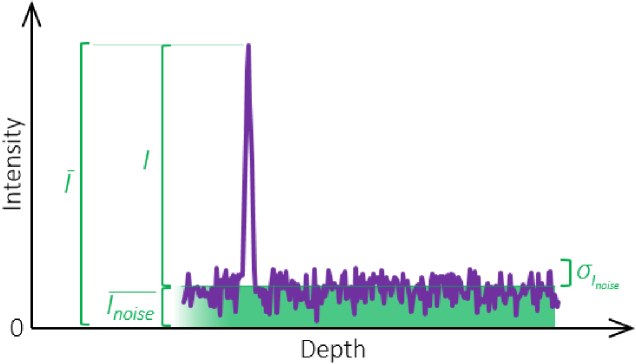
Intensity signal and noise background in a schematic OCT depth profile. The noise floor is characterized by its average intensity $\bar{I_{noise}}$ and its variance $\sigma_{I_{noise}}^{2}$. The measured OCT intensity signal $\bar{I}$ consists of the pure signal intensity I biased by the average noise level $\bar{I_{noise}}$.

The signal-to-noise ratio is the measure of choice to describe the influence of noise on signal measurements. An overview of methods for determining the SNR and in particular the sensitivity of an OCT system has recently been published by Agrawal et al. [24]. The SNR in OCT is typically defined as the ratio of the average signal intensity to the standard deviation of the noise intensity, $\text{SNR}=\bar{I}/\sigma_{I_{noise}}$ [24–28] (or alternatively $\bar{\left\langle I\right\rangle }/\langle \sigma_{I_{noise}}\rangle$ for averaged signals). As shown in the previous sections and visualized in Fig. 2

, the measured signal intensity $\bar{I}$ is the sum of the pure signal intensity I and the noise floor $\bar{I_{noise}}$. Historically, the bias caused by the noise floor is not specifically factored out from the SNR analysis, however some investigations of OCT image data did exclude the background [29,30]. Because of the multiple approaches for OCT data processing, it makes sense to ask, "how important is noise bias correction when measuring SNR?". In the following section, we will investigate the SNR for the total measured signal (i.e. including the contribution of the noise floor) with and without averaging. Subsequently, we will account for the signal offset caused by the noise floor and evaluate the SNR with noise bias correction.

#### 2.3.1 Signal-to-noise performance without noise bias correction

Using the signal and noise pairs in Eqs. (13) and (12), (18) and (17), and (22) and (21), and the relation $\bar{I_{noise}}=\sigma_{I_{noise}}=2\sigma^{2}$ in Eqs. (11) and (12), we find the following SNRs for single signals, N magnitude averaged signals, and N complex averaged signals, respectively:

(24) $${\text{SNR}}_{1}=\frac{\bar{I}}{\sigma_{I_{noise}}}=\frac{I+\bar{I_{noise}}}{\sigma_{I_{noise}}},$$

(25) $${\text{SNR}}_{MAG}=\frac{\bar{\langle I\rangle_{MAG}}}{\langle \sigma_{I_{noise}}\rangle_{MAG}}=\frac{I+\bar{I_{noise}}}{\frac{1}{\sqrt{N}}\sigma_{I_{noise}}}=\sqrt{N}\cdot {\text{SNR}}_{1},$$

(26) $${\text{SNR}}_{CPX}=\frac{\bar{\langle I\rangle_{CPX}}}{\langle \sigma_{I_{noise}}\rangle_{CPX}}=\frac{I+\frac{1}{N}\bar{I_{noise}}}{\frac{1}{N}\sigma_{I_{noise}}}=N\cdot {\text{SNR}}_{1}-(N-1).$$

In short, averaging the magnitudes of N signals improves the SNR by a factor $\sqrt{N}$ whereas complex averaging of N signals changes the SNR by a factor N less (N−1).

**Fig. 3.**
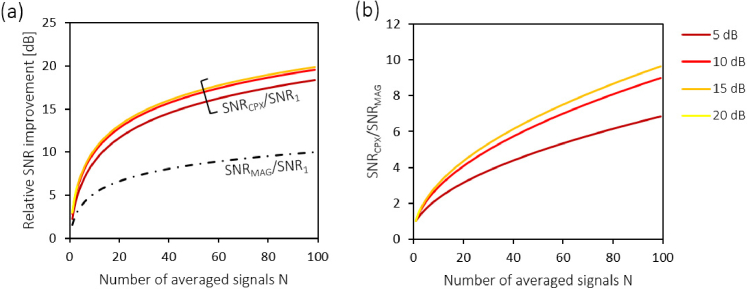
Relative SNR improvement by signal averaging for strong input signals (without noise bias correction). (a) The ratios SNR_*CPX*_/SNR_1_ and SNR_*MAG*_/SNR_1_ are shown for N from 1 to 100. SNR_*MAG*_/SNR_1_ is plotted as a dash-dotted line, whereas the SNR_1_-dependent ratio SNR_*CPX*_/SNR_1_ is plotted in rainbow colors for several SNR_1_ values between 5 dB and 50 dB. Note that SNR_*CPX*_/SNR_1_ converges to an *N*-fold improvement. (b) The ratio ${\text{SNR}}_{CPX}/{\text{SNR}}_{MAG}=\left(N-(N-1)/{\text{SNR}}_{1}\right)/\sqrt{N}$ is plotted for the spectrum of SNR_1_ values used in (a). Note that in particular for strong input signals with high SNR_1_, complex averaging outperforms magnitude averaging and converges to a $\sqrt{N}$-fold better SNR performance. As the SNR profiles converge for large SNRs, the curves in (a) and (b) start to overlap for values greater than 15 dB.

The relative SNR improvements SNR_*MAG,CPX*_/SNR_1_ and SNR_*CPX*_/SNR_*MAG*_ are plotted for *N* up to 100 and for typical OCT image dynamics with SNR_1_ between 5 dB and 20 dB in Fig. 3

. In particular for small N, complex averaging provides a considerable SNR improvement over magnitude averaging. At the same time, the SNR_1_ dependence of complex averaging has a considerable impact on its performance: While for strong signals with SNR_1_ ≫ 1, complex averaging outperforms magnitude averaging by a factor $\sqrt{N}$, the signal-to-noise improvement becomes much less for weaker input signals.

In the next section, we are going to explore the SNR enhancement of the two averaging approaches particularly for very weak signals on the order of the noise background, however still without correcting for the signal bias caused by the noise floor. Will magnitude averaging or complex averaging be superior in terms of recovering such small signals?

#### 2.3.2 Averaging domains and borderline SNR

When the signal bias caused by the noise is not accounted for, the SNR performance averaging depends on the approach taken. Equations (25) and (26) revealed a dependence on the number of averaged signals, N, and – for complex averaging – on the SNR of the input signals, SNR_1_. In this section, we are investigating this dependence for different numbers of averaged signals, N, and for a great range of signals – from much smaller than the noise variance to several orders of magnitude greater. Recalling the identities for the noise variance and the observed signal intensity in Eqs. (12) and (13), we choose the quantity $I/2\sigma^{2}$, which is identical to SNR_1_ − 1, as the benchmark for the input signal.

Figure 4(a)

**Fig. 4.**
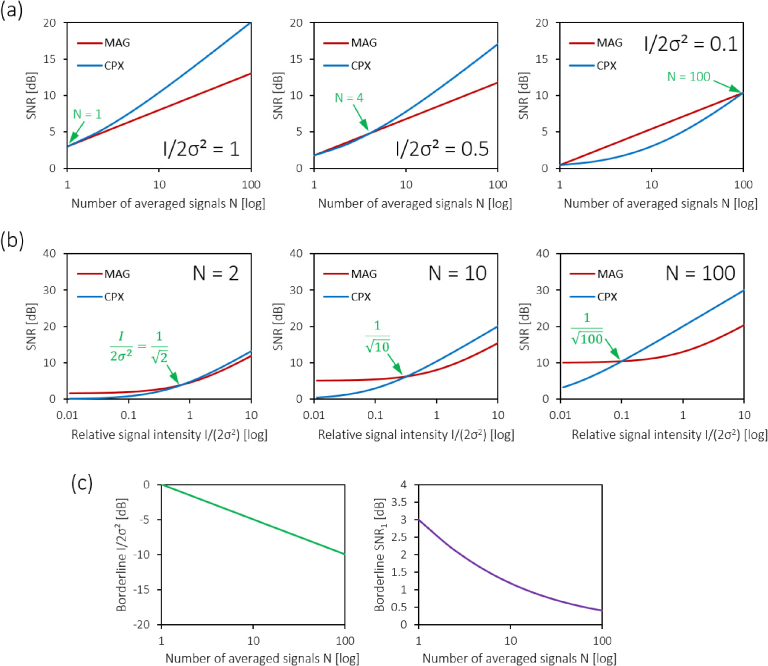
Influence of input signal level and number of averaged signals on the SNR (without noise bias correction). (a) SNR_*MAG*_ and SNR_*CPX*_ after magnitude and complex averaging of N signals plotted for relative signal levels of $I/2\sigma^{2}=1$ (left), $I/2\sigma^{2}=0.5$ (middle), and $I/2\sigma^{2}=0.1$ (right), respectively. (b) SNR_*MAG*_ and SNR_*CPX*_ after magnitude and complex averaging of signals $I/2\sigma^{2}$ ranging from 0.01 through 10, plotted for averages of N=2 (left), N=10 (middle), and N=100 signals (right), respectively. Green arrows in (a) and (b) indicate the intercepts of the SNR profiles, i.e. the borderline SNR where magnitude and complex averaging perform equally. (c) Borderline plots of $I/2\sigma^{2}=1/\sqrt{N}$ as well as SNR_1_ as described in Eq. (27).

shows three plots of the signal-to-noise ratios calculated for magnitude averaging (red) and complex averaging (blue) where relatively small signals $I/2\sigma^{2}=1$, 0.5 and 0.1 were chosen as the respective inputs. Note that for $I=2\sigma^{2}$ (left plot), complex averaging always yields greater SNR than magnitude averaging for N>1. However, for $I/2\sigma^{2}<1$ and a small number of signals N, magnitude averaging provides a more effective SNR improvement. Figure 4(b) plots three pairs of SNR profiles for a fixed number of averaged signals (N=2, 10 and 100), this time as a function of the relative signal intensity $I/2\sigma^{2}$. Again, for weak input signals, the superior performance of magnitude averaging can be observed, while complex averaging excels beyond a crossover point of $I/2\sigma^{2}=1/\sqrt{N}$. This relative borderline signal is plotted for N up to 100 in Fig. 4(c) alongside the borderline input SNR_1_

(27) $${\text{SNR}}_{1,borderline}=1+\frac{1}{\sqrt{N}}$$

which is the input SNR_1_ for which magnitude averaging and complex averaging perform equally well.

**Fig. 5.**
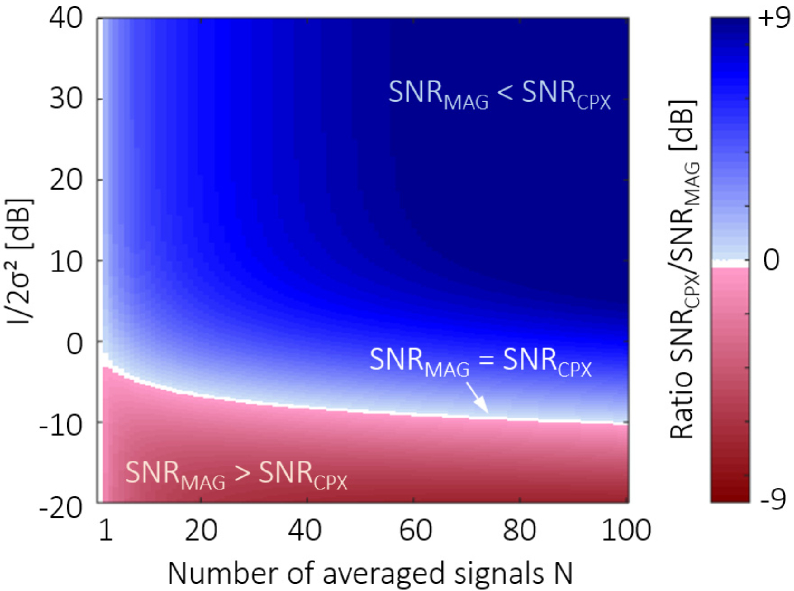
Relative SNR performance for magnitude and complex averaging (without noise bias correction). The heat map displays the ratio SNR_*CPX*_/SNR_*MAG*_ in decibels for relative input signals $I/2\sigma^{2}$ ranging from -20 dB to +40 dB and up to a number of averaged signals N=100. For small input signals below the noise level, magnitude averaging yields a better SNR improvement (red range), while complex averaging performs better for greater N and stronger input signals (blue range). The borderline SNR where SNR_*MAG*_ = SNR_*CPX*_ separates these two domains (white plot).

An overview of the relative SNR performance SNR_*CPX*_/SNR_*MAG*_ at various inputs $I/2\sigma^{2}$ and N is shown as a heat map in Fig. 5

. At first glance, two domains can be differentiated: For stronger signals $I/2\sigma^{2}$ and larger N, complex averaging provides a greater SNR improvement than magnitude averaging (see the area in blue in Fig. 5). For signals comparable to or smaller than the noise level, magnitude averaging yields a better SNR enhancement (see the area in red). In between these two domains, the borderline with equal SNR performance of the two approaches is indicated in white, SNR_*CPX*_/SNR_*MAG*_ = 1.

#### 2.3.3 SNR in absence of a signal (noise floor only)

An interesting scenario is posed by the calculation of the SNR when the signal intensity I is zero. Then, using the relation $\bar{I_{noise}}=\sigma_{I_{noise}}=2\sigma^{2}$ from Eqs. (11) and (12), the SNRs for a single signal, after magnitude averaging and after complex averaging, respectively, become:

(28) $${\text{SNR}}_{1}(I=0)=\frac{\bar{I_{noise}}}{\sigma_{I_{noise}}}=1,$$

(29) $${\text{SNR}}_{MAG}(I=0)=\frac{\bar{I_{noise}}}{\frac{1}{\sqrt{N}}\sigma_{I_{noise}}}=\sqrt{N},$$

(30) $${\text{SNR}}_{CPX}(I=0)=\frac{\frac{1}{N}\bar{I_{noise}}}{\frac{1}{N}\sigma_{I_{noise}}}=1.$$

An obvious disunity of the SNRs calculated for pure noise signal can be observed. This calls for a revised definition of the SNR, this time accounting for the signal bias $\bar{I_{noise}}$ imposed by the noise floor.

#### 2.3.4 SNR analysis with noise bias correction

In the limit of a very weak OCT signal, the small meaningful signal contribution sits on top of a comparatively large noise floor. This noise bias is evident as the term related to $\bar{I_{noise}}$ in Eqs. (24–26) and is also visualized in Fig. 2. Thus, it makes sense to actually consider the impact of this noise bias in the SNR analysis – in particular for weak signals. In this section, we introduce and evaluate an SNR analysis with noise bias correction. A similar approach has for instance also been used by Makita et al. [39] and recently been modified by our group [40] to correct for noise contributions in PS-OCT images and improve the computation of the degree of polarization uniformity for weak signals. To some extent, this SNR with noise bias correction resembles the SNR definitions in Refs. [29,30] and has formal similarities with previous definitions of the contrast-to-noise ratios in Refs. [21,27].

The noise bias of the average OCT signals can be removed by subtracting the average noise intensity ($\bar{I_{noise}}$, $\bar{\langle I_{noise}\rangle_{MAG}}$, and $\bar{\langle I_{noise}\rangle_{CPX}}$ in Eqs. (11), (16) and (20), respectively) from the average signal intensity ($\bar{I}$, $\bar{\langle I\rangle_{MAG}}$, and $\bar{\langle I\rangle_{CPX}}$ in Eqs. (13), (18) and (22), respectively). The noise bias corrected average signal intensities then read

(31) $${\bar{I}}^{\prime }=\bar{I}-\bar{I_{noise}}=I+2\sigma^{2}-2\sigma^{2}=I,$$

(32) $${\bar{\langle I\rangle_{MAG}}}^{\prime }=\bar{\langle I\rangle_{MAG}}-\bar{\langle I_{noise}\rangle_{MAG}}=I+2\sigma^{2}-2\sigma^{2}=I,$$

(33) $${\bar{\langle I\rangle_{CPX}}}^{\prime }=\bar{\langle I\rangle_{CPX}}-\bar{\langle I_{noise}\rangle_{CPX}}=I+\frac{1}{N}2\sigma^{2}-\frac{1}{N}2\sigma^{2}=I.$$

Note that the three corrected average signal intensities now match the pure signal intensity I. Further, using the noise variances in Eqs. (12), (17) and (21), the respective noise bias corrected SNRs can be calculated as

(34) $$SNR_{1}^{\prime }=\frac{{\bar{I}}^{\prime }}{\sigma_{I_{noise}}}=\frac{I}{2\sigma^{2}},$$

(35) $$SNR_{MAG}^{\prime }=\frac{{\bar{\langle I\rangle_{MAG}}}^{\prime }}{\langle \sigma_{I_{noise}}\rangle_{MAG}}=\sqrt{N}\frac{I}{2\sigma^{2}}=\sqrt{N}\cdot SNR_{1}^{\prime },$$

(36) $$SNR_{CPX}^{\prime }=\frac{{\bar{\langle I\rangle_{CPX}}}^{\prime }}{\langle \sigma_{I_{noise}}\rangle_{CPX}}=N\frac{I}{2\sigma^{2}}=N\cdot SNR_{1}^{\prime }.$$

**Fig. 6.**
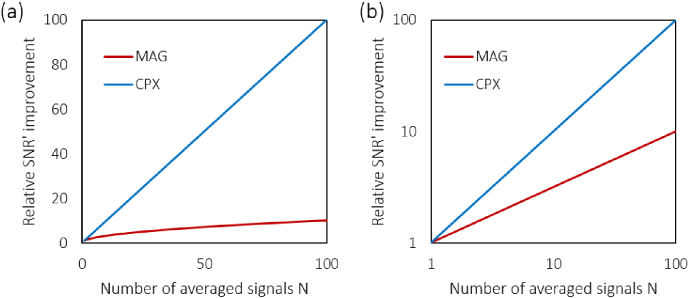
Theoretical improvement of the signal-to-noise ratio SNR^′^ after noise bias correction plotted on (a) linear scales and (b) log scales. A $\sqrt{N}$- and *N*-fold improvement of the SNR^′^ of a single signal can be observed for magnitude and complex averaging, respectively. Note that, unlike for the noise-afflicted SNR calculations in Figs. 3 through 5, neither of the averaging approaches depend on the input signal strength.

$SNR_{1}^{\prime }$ equates the quantity $SNR-1=I/2\sigma^{2}$ already known from the analyses in the previous sections. Even more strikingly, the noise bias corrected SNRs after magnitude and complex averaging now exhibit a simple proportionality of $\sqrt{N}$ and N with $SNR_{1}^{\prime }$ (Fig. 6

), which also means that SNR*′* will be zero if there is no signal for Eqs. (34–36). Using the noise bias correction, complex averaging thus always yields a $\sqrt{N}$-times better SNR than magnitude averaging, even for weak input signals and small N.

## 3. Experimental validation

### 3.1 Numerical simulation

OCT signals and noise were simulated according to the probability density functions described in Eqs. (3) and (6), respectively. For the noise contribution, binormally distributed complex signals with similar standard deviations σ along both the real axis and the imaginary axis were generated. For the signal contribution, the binormal distribution was generated around a real-valued signal A such that the phasor distribution was shifted from the origin of the complex plane to (A,0) (see also Fig. 1(e)). A total of M=100 averaged signals were computed for every simulation run. For complex averaging, in every run N=1 to N=100 complex-valued phasors were averaged. For magnitude averaging, the absolute values were computed for every single noise and signal phasor prior to averaging. Finally, the respective SNRs and SNR^′^s were calculated from the average signal intensity and the variance of the noise signals for every N.

**Fig. 7.**
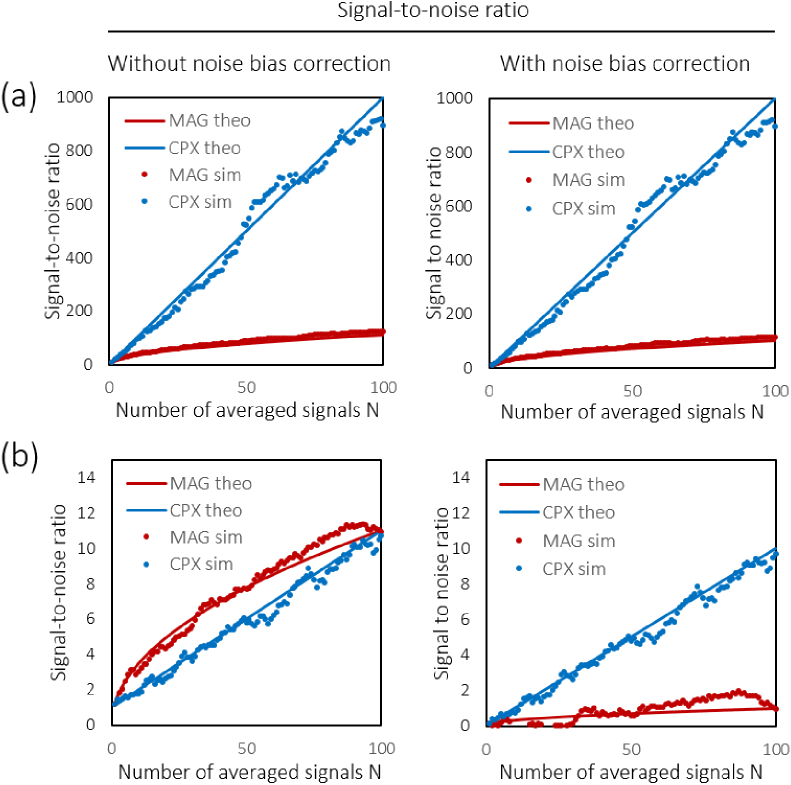
Simulation of the effect of averaging N signals with relative strength $I/2\sigma^{2}=10$ in (a) and $I/2\sigma^{2}=0.1$ in (b). Shown are the averaged signal-to-noise ratios calculated from N simulated phasors (∙) alongside the corresponding theoretical plots (−). The SNRs without and with noise bias correction are plotted in the left and right panels, respectively. Without noise bias correction, the SNR_*CPX*_ shows a better performance for the strong signal in (a), whereas SNR_*MAG*_ dominates for N=1 to 100 both for theoretical calculation and simulation. With noise bias correction (rightmost column), complex averaging similarly provides an *N*-fold improvement of the respective SNR^′^ = *I*/2*σ*^2^ whereas an $\sqrt{N}$-fold improvement can be observed for magnitude averaging.

Results of the simulations performed in MATLAB (R2014a, MathWorks) are shown in Fig. 7

. To showcase the effect of averaging for strong and weak signals, SNR simulations for two distinct relative signal levels of $I/2\sigma^{2}=10$ and $I/2\sigma^{2}=0.1$ are presented in Fig. 7(a) and (b), respectively. Unlike magnitude averaging, complex averaging markedly decreased the signal intensity but at the same time also reduced noise much more by averaging. For the standard deviation of the noise fluctuations, a decrease by $1/\sqrt{N}$ and 1/N was observed for magnitude and complex averaging, respectively. The resulting SNRs are shown in the four panels in Fig. 7. Here, for averaging up to 100-fold, SNR_*CPX*_ reveals a better SNR improvement for the strong input signal (Fig. 7(a)) when the noise bias is not corrected for, while magnitude averaging appears more effective for the weaker input signal in Fig. 7(b). After noise bias correction, however, no more dependence upon the input signal level can be observed, and complex averaging provides an *N*-fold increase in SNR^′^ whereas the SNR^′^ is only enhanced by a factor $\sqrt{N}$ after magnitude averaging.

### 3.2 Averaging experimental OCT data

For demonstrating the effect of the different averaging approaches on OCT images, we used a spectral domain (SD) OCT system in our lab [38]. This polarization sensitive SD-OCT system was used for imaging a stationary eye phantom. This system is based on a multiplexed superluminescent diode (*λ* = 840 nm, Δ*λ* = 100 nm) as a light source, a free-space Michelson-type interferometer, and a polarization-sensitive detection unit including two spectrometers. For the investigations presented here, only the co-polarized detection channel was analyzed; thus the PS-OCT system was reduced to what would be considered a standard SD-OCT with high-resolution imaging capabilities - 3.6 *μ*m axial resolution (assuming a refractive index of 1.35), 83 kHz A-scan rate - similar to state-of-the-art commercial SD-OCT scanners for retinal imaging.

**Fig. 8.**
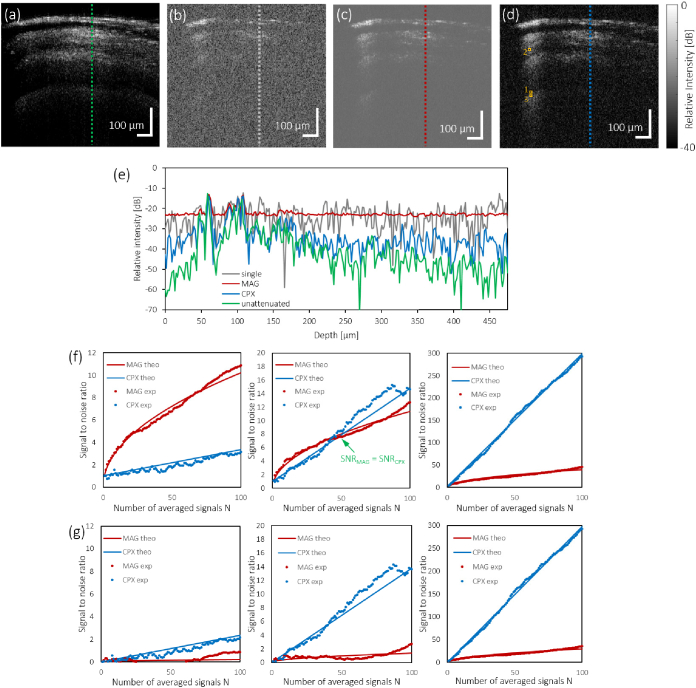
Experimental verification of SNR improvement by the different averaging approaches in a layered retina phantom. (a) Single B-scan image of the phantom (no attenuation). (b) Single B-scan image after attenuating the sample beam by 30 dB. (c) B-scan image after averaging the magnitudes of 100 repeated frames (with 30 dB attenuation). (d) B-scan image after averaging the phasors of 100 repeated frames (with 30 dB attenuation). Note that all B-scan images in (a-d) are displayed with identical dynamic ranges of 40 dB where 0 dB refers to the maximum signal intensity in the frame. (e) Depth profiles of a single A-scan before and after attenuation, 100 magnitude averaged, and 100 complex averaged A-scans at the locations indicated by the dotted lines in (a-d). Due to a beam offset caused by the ND filter, the scattering profile of the unattenuated case has a slightly different structure. Dynamic range as in (a-d). (f) SNR improvement without noise bias correction for three pixels with weak (left), borderline (middle) and strong signal strength $I/2\sigma^{2}$ (right), respectively. Pixel locations are indicated by orange boxes numbered with 1-3 in panel (d). SNR curves are shown for magnitude and complex averaging of 1-100 repeated M-scan signals for the experimental data (∙) alongside the corresponding theoretical plots (−). (g) SNR^′^ improvement after noise bias correction for the data sets shown in panel (f). Note that the experimental data (∙) slightly fluctuates around the theoretical profiles (−). SNR^′^ data fluctuating below SNR^′^ = 0 is not shown. The axes are scaled as in the respective plots in (f) in order to enable a direct comparison between the two SNR analyses.

The eye phantom, composed of several layers of transparent nail polish on a glass bead to produce a laminar reflectance pattern [38], was imaged using B-scan and M-scan protocols at different levels of attenuation (from 0 dB to -40 dB) to observe the effects of averaging in low-signal conditions. The B-scan protocol scanned a 1 mm lateral range with 100 repeats to generate cross-sectional images of the phantom (Fig. 8(a-d)

). Representative depth profiles are shown in Fig. 8(e).

The M-scan protocol was used to acquire 1000 repeated A-lines from a fixed position within the phantom for a precise comparison of averaging methods. The M-scan protocol was chosen to minimize phase differences between consecutive A-lines and ensure that averaging represents a best-case real-world scenario. The 1000 A-lines were split into 10 temporally independent bins of 100 consecutive A-lines each. Complex and magnitude averaging for N up to 100 was performed on each of the 10 bins and the resulting signal curves were averaged together before calculating SNR and SNR^′^ in order to yield more stable SNR curves, particularly for lower numbers of averages. Here, the noise variances were computed from the noise pixel data of 100 consecutive A-lines, similar to the simulation above. Three characteristic SNR and SNR^′^ curves from the magnitude-averaging dominant, borderline, and complex-averaging dominant domains are shown in Fig. 8(f) and (g). Theoretical SNR profiles based on SNR_1_ as described in Eqs. (25) and (26) demonstrate good agreement with the experimental data in Fig. 8(f). These results show that the theoretical magnitude-averaging dominant domain is reproducible with real world OCT data when the noise floor induced bias is not accounted for. Similarly, the SNR^′^ plots from the experimental data with noise bias correction follow the expected slopes of $\sqrt{N}$ and N (Fig. 8(g)).

## 4. Discussion and conclusion

Signal averaging is one of the most commonly used procedures for OCT image processing. Here, we investigated the SNR performance of magnitude and complex averaging in theory and backed our findings with experimental results from simulations and actual OCT image data. We also studied the effect of the background noise bias on the measured SNR and calculated a noise bias corrected SNR. In the following paragraphs, the main observations are summarized and discussed in order to provide a better understanding of the strengths and weaknesses of the two approaches.

### 4.1 Impact of averaging on noise and signals

Complex averaging reduces the noise variance by 1/N, and thus provides a much stronger noise reduction than magnitude averaging, which only reduces the noise by $1/\sqrt{N}$. At the same time, complex averaging also reduces the signal – in contrast to magnitude averaging, which maintains the signal level and only reduces noise. The main cause of the average signal decrease by complex averaging is the reduction of the average noise level. Both signal and noise characteristics have to be considered to understand the impact of averaging on OCT data.

### 4.2 Leaving or removing the noise floor bias for the SNR calculation

The signal-to-noise ratio is a commonly used measure to assess the image quality and to describe the system performance of OCT machines. Usually, the ratio of the average intensity of a signal peak and the standard deviation of the noise intensity is used to calculate the SNR [24,28]. While it is rather clear how to estimate the noise variance (e.g., by considering the noise intensity in a signal-free image area), the definition of the average intensity is not that obvious. As visualized in Fig. 2, taking the entire measured signal intensity from the zero line to the (average) peak intensity $\bar{I}$ includes two portions, namely the actual signal intensity term I and the background noise level $\bar{I_{noise}}$.

For relatively strong signals, the measured signal is dominated by the intensity term I and $\bar{I}\approx I\gg \bar{I_{noise}}$. In contrast, when the actual signal contribution to the measured signal is small (i.e. $I\ll \bar{I}$), the noise floor bias $\bar{I_{noise}}$ prevails. We hence investigated the signal-to-noise performance of magnitude and complex averaging for (a) leaving the noise floor bias as part of the measured signal and (b) correcting for the noise bias. As discussed in detail in the following sections, similar results were observed for both SNR analyses when strong signals were investigated. However, for rather weak signals, the impact of $\bar{I_{noise}}$ manifested in the observation of dissimilar SNR characteristics.

### 4.3 Noise-afflicted signal-to-noise ratios after averaging strong and weak signals

For sufficiently large signals $\bar{I}=I+\bar{I_{noise}}$, the $N\cdot {\text{SNR}}_{1}$ term in Eq. (26) dominates such that complex averaging converges on an *N*-fold SNR improvement, while magnitude averaging only enhances the SNR $\sqrt{N}$-fold. In contrast, for recovering weak signals comparable to or smaller than the noise, magnitude averaging appeared to outperform complex averaging – in particular for small N. When the input SNR_1_ was just slightly larger than unity (i.e., $I/2\sigma^{2}$ just slightly greater than zero), the right-hand side of Eq. (26) was essentially neutralized such that the SNR improvement was far less than the $\sqrt{N}$-fold enhancement yielded by magnitude averaging, especially in the limit of small N. Hence, magnitude averaging could be considered more effective for boosting small signals as they may be found in the outer nuclear layer in the retina when the noise floor induced signal bias is not accounted for. However, by taking a look at the SNR in absence of an actual signal (I=0), odd SNR characteristics were observed (see section 2.3.3) which underscored the importance of a noise bias correction.

### 4.4 SNR calculations with noise bias correction

When SNRs are calculated in the classical way described in section 2.3.1, the average noise floor level $\bar{I_{noise}}$ contributes to the intensity measured as "signal". This noise bias impacts the measured SNR, in particular for weak signals. Akin to noise offset removal in PS-OCT processing [39,40], we performed SNR calculations with noise bias correction in sections 2.3.4 and 3. By using this modified approach, a superior SNR performance was always observed for complex averaging, regardless of the input signal strength. Compared to the noise bias corrected SNR of a single signal prior to averaging, magnitude and complex averaging improved the SNR (after noise bias correction) by a factor of $\sqrt{N}$ and N, respectively. The theoretically predicted performance agreed well with the performance observed in simulated and experimental OCT data (Figs. 7 and 8). This finding suggests that noise bias correction definitely is an important processing step in SNR analyses – especially when it comes to averaging weak signals.

### 4.5 Implications for practical OCT image averaging

State-of-the-art swept source and spectral domain OCT devices deliver complex-valued signals (see Eq. (1)) right out of the box. Hence both magnitude and complex averaging can be easily implemented using the image data. For effective complex averaging, it is imperative that the phases of the signals are accurately aligned before averaging as described in more detail in Appendix B. While this means additional computational steps, it may be well worth the effort when the SNR is to be increased in images of scattering structures such as the retina where OCT has been frequently applied. Retinal OCT images may easily span a dynamic range of 40 dB with hyperscattering structures such as the nerve fiber layer and the pigment epithelium that can serve as landmarks for phasor alignment [18]. Other retinal tissues such as the ganglion cell layer and the outer nuclear layer [41], and also structures in the vitreous, which are usually much less reflective [42], may then be visualized by averaging multiple OCT images together. Complex averaging has also been found promising to detect signals in settings with multiple scattering [20].

Magnitude averaging on the other hand can be implemented at less computational expense and may be the averaging approach of choice for images containing mostly weak signals of interest, which would render phase alignment for complex averaging difficult or even impossible. Also in scenarios where images include a lot of varying motion, e.g. when visualizing weakly scattering structures such as flow in lymphatic vessels [43], albeit theoretically less effective by $\sqrt{N}$ in terms of SNR improvement, magnitude averaging may likely trump complex averaging.

### 4.6 Future perspectives

Finally, we would like to point out that, while the analysis presented here particularly focused on the SNR improvement for different averaging approaches, it may be interesting to also investigate other image metrics such as their contrast-to-noise characteristics and/or their efficiency in terms of speckle reduction. For this purpose and to explore scenarios with tissue-like scattering, recently described OCT signal models could be particularly interesting candidates [28,44–46]. Additionally, OCT images are often displayed on a logarithmic scale in order to compress signals covering a large dynamic range. The logarithm pulls up weak signals but also the noise floor [21], such that the SNR performance for averaged logarithmic amplitude data will deviate from that for uncompressed OCT data discussed here. An in-depth analysis similar to the one performed for linear data in section 2.2 may provide more insight in the advantages and disadvantages of magnitude- and phasor-based averaging approaches for enhancing OCT image quality.

## Figures and Tables

**Fig. 9.**
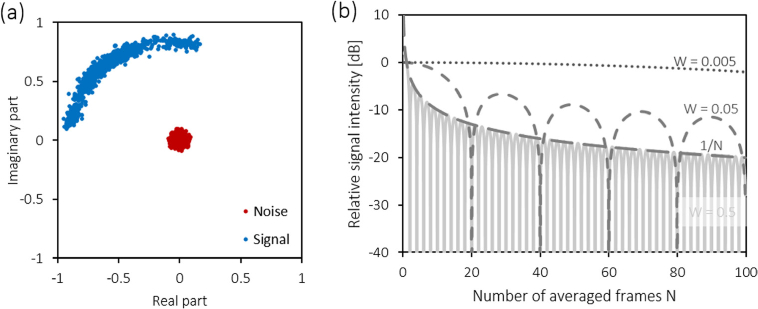
Phasor rotation by axial motion and signal penalty after complex averaging of axially displaced signals. (a) Example of a signal phasor trajectory of 1000 repeated measurements of a weak reflector (an attenuated glass surface). Over 1/70 second, the phasor (blue dots) is markedly moving around the origin. Corresponding noise signals are shown as red dots. The main source of displacement in this measurement was air flow from a nearby air condition outlet. (b) Relative signal intensity decrease caused by complex averaging of N signals with relative displacements W of 0.5 (solid line), 0.05 (dashed line), and 0.005 (dotted line). For reference, 1/N is plotted as well. While relative displacements of *λ*/200 (*W* = 0.005) between consecutive signals almost do not affect the intensity of the averaged signal, greater displacements such as λ/20 and λ/2 cause a periodic, significant signal reduction. In unlucky cases, P(N,W) reaches zero such that the averaged signal is completely annihilated.

## Appendix

### Appendix A – Glossary

The table below provides an overview of the variables and symbols used in this article.

**Table 1.** Overview of variables and abbreviations

Symbol	Explanation	Equation
A(x,t)	Amplitude of phasor representing the OCT signal	(1)
$\bar{A}$	Mean signal amplitude	(7)
$A_{noise}$	Amplitude of complex phasor representing the OCT noise signal	(3)
$\bar{A_{noise}}$	Mean noise amplitude	(4)
Δϕ	Phase difference	(39,42)
I	Signal intensity, $I=A^{2}$	
$\bar{I}$	Mean signal intensity	(13)
${\bar{I_{x}}}^{\prime }$	$\bar{I_{x}}$ with noise bias correction, (*x* = void,*MAG,CPX*)	(31–33)
$I_{noise}$	Noise intensity, $I_{noise}=A_{noise}^{2}$	
$\bar{I_{noise}}$	Mean noise intensity	(11)
$i_{noise}$	Imaginary part of complex phasor representing the OCT noise signal	(2)
N	Number of averaged signals	
$p_{A}(A_{noise})$	PDF describing amplitudes of random phasors (Rayleigh distribution)	(3)
$p_{\Delta \phi }(\Delta \phi ,A)$	PDF describing distribution of phase differences	(43)
$p_{I}(I_{noise})$	PDF describing intensities of random phasors	(9)
$p_{I}(I,I_{noise})$	PDF describing intensities of signals in presence of noise	(10)
$p_{A}(A,A_{noise})$	PDF describing amplitudes of signal phasors in presence of noise (Rice distribution)	(6)
$p_{ri}(r_{noise},i_{noise})$	Binormal PDF describing random phasors	(2)
P(N)	Penalty factor after averaging N signals	(37)
PDF	Probability density function	(2,3,6,9,10)
ϕ(x,t)	Phase of phasor representing the OCT signal	(1)
$r_{noise}$	Real part of complex phasor representing the OCT noise signal	(2)
$S_{OCT}(\text{x},t)$	Complex-valued OCT signal	(1)
$S_{OCT}^{\left(moco\right)}$	OCT signal after motion correction (moco)	(40,41,45)
$\sigma^{2}$	Variance of real/imaginary part of $p_{ri}$	(1)
$\sigma_{A}^{2}$	Variance of signal amplitudes for $p_{A}(A,A_{noise})$	(8)
$\sigma_{I}^{2}$	Variance of signal intensity for $p_{I}(I,I_{noise})$	(14)
$\sigma_{I_{noise}}^{2}$	Variance of noise intensity for $p_{I}(I_{noise})$	(12)
$\sigma_{noise}^{2}$	Variance of noise amplitudes for $p_{A}(A_{noise})$	(5)
$SNR_{1}$	SNR of non-averaged signal	(24)
$SNR_{1,borderline}$	SNR_1_ of non-averaged signal for $SNR_{MAG}=SNR_{CPX}$	(27)
$SNR_{MAG}$	SNR after magnitude averaging	(25)
$SNR_{CPX}$	SNR after complex phasor averaging	(26)
$SNR_{x}^{\prime }$	$SNR_{x}$ with noise bias correction, (x=1,MAG,CPX)	(34–36)
$\langle \cdot \rangle_{MAG}$	Quantity ⋅ after magnitude averaging	(16–19)
$\langle \cdot \rangle_{CPX}$	Quantity ⋅ after complex averaging	(20–23)

### Appendix B – Complex signal averaging in case of axial motion

#### Effect of axial motion on complex averaging

Keeping the signal phase ϕ constant is essential for complex signal averaging. If ${\text{d}}^{2}\phi (x,t)/\text{d}x\text{d}t\neq 0$, the complex sum of the signal vectors will have a length smaller than the sum of their absolute values. In other words, if the phases of the complex signals are not matched prior to averaging, the length of the average signal vector will be reduced. The condition Δϕ=0 is fulfilled for simultaneously acquired signals at the same *z*-position within the same speckle. When averaging signals among different speckles acquired at the same time point, first the phase offset between different speckles has to be eliminated [48]. Similarly, when signals acquired at the same location x but at different time points t are averaged (e.g., signals from repeatedly acquired OCT images), the influence of axial motion has to be removed prior to averaging [18].

Axial motion introduces a proportional phase shift in the complex signals (Fig. 9(a)). This phase shift has been extensively exploited for velocity measurements in Doppler OCT, for displacement measurements in OCT elastography and, recently most prominently, for some OCT angiography approaches [32–36]. The phase shift of an OCT signal is proportional to 2kΔz where k=2π/λ is the central wavenumber and Δz is the axial displacement between successive measurements. When N complex signals with displacements $\Delta z_{j}$ relative to the first signal are averaged in a complex fashion, the resulting relative signal reduction corresponds to the penalty factor P(N):

(37) $$P(N)=\frac{1}{N}\left|\sum_{j=1}^{N}e^{i2\pi W_{j}}\right|$$

where $W_{j}=2\Delta z_{j}/\lambda$ denotes the displacement of the *j*-th signal in terms of wavelengths. In case of constant axial motion, the displacement will increase by (j−1)Δz for the *j*-th signal compared to the first signal. The penalty factor P(N) when averaging N signals with relative separation $W_{j}=W$ is

(38) $$P(N,W)=\frac{1}{N}\left|\sum_{j=1}^{N}e^{i2\pi Wj}\left|=\frac{1}{N}\right|\frac{\sin[\pi W(N+1)]}{\sin[\pi W]}e^{i\pi NW}-1\right|.$$

Examples for the relative signal intensity decrease due to P(N,W) are shown in Fig. 9(b). For large W, the upper limit of the rectified sinc-like decrease is given by 1/N.

#### Compensation of axial motion

In order to remove the detrimental effect of axial motion and thus to fully exploit the SNR advantage of complex averaging, the phases $\phi_{j}$ of the signals to be averaged have to be aligned. Ju et al. proposed two approaches: one compensating phase offsets A-line-wise and another one compensating phase offsets in a pixel-wise fashion [18]. Assuming a constant "bulk" displacement of the simultaneously acquired signals within one A-scan, the bulk phase shift $\Delta \phi_{bulk}$ between the *m*-th A-line in a reference frame (*ref*) and the corresponding *m*-th A-scan in other frames to be averaged can be calculated by computing the angle of the weighted complex signal average along every A-scan:

(39) $$\begin{array}{rl} \Delta \phi_{bulk}^{\left(m\right)} & =\text{arg}\;\left[\sum_{z=z_{0}}^{z_{max}}\left(A_{m}^{j}(z)e^{i\phi_{m}^{j}(z)}\right)\;{\left(A_{m}^{ref}(z)e^{i\phi_{m}^{ref}(z)}\right)}^{*}\right]=\\ & =\text{arg}\;\left[\sum_{z=z_{0}}^{z_{max}}A_{m}^{j}(z)A_{m}^{ref}(z)e^{i[\phi_{m}^{j}(z)-\phi_{m}^{ref}(z)]}\right] \end{array}$$

where m, j, and ∗ denote the A-scan index, the B-scan index and the conjugate complex, respectively, while $z_{0}$ and $z_{max}$ are the axial depth limits. By subtracting the bulk phase shift vector $\Delta \phi_{bulk}^{\left(m\right)}$ from the phases of every transverse line of the OCT image, an axial motion corrected (moco) image is generated:

(40) $$S_{OCT}^{\left(moco\right)}(x,z)=A_{m}^{j}(x,z)\exp\;\left[i(\phi_{m}^{j}(x,z)-\Delta \phi_{bulk}^{\left(m\right)})\right].$$

The assumption that displacements are constant along the depth profile does however not hold for all samples. In particular live biological samples are subject to dynamic deformations, which may for instance be caused by pulsating blood vessels. Hence, in another complex image averaging approach, all complex signals of one reference B-scan serve as a 2D baseline image $S_{OCT}^{\left(ref\right)}(x,z)$ [18]. The phasors in all pixel locations (x,z) of the subsequent frames are then aligned with respect to those in the reference frame by multiplying them to the conjugate complex of $S_{OCT}^{\left(ref\right)}(x,z)$ in an amplitude weighted manner,

(41) $$S_{OCT}^{\left(j,moco\right)}(x,z)=\sqrt{A_{j}(x,z)A_{ref}(x,z)}\exp\;\left[i\;(\phi_{j}(x,z)-\phi_{ref}(x,z))\right].$$

Since Eq. (41) does not only align phasors at signal locations but also all noise phasors, complex averaging of $S_{OCT}^{\left(j,moco\right)}(x,z)$ essentially corresponds to magnitude averaging. This detriment can be overcome by locally averaging the phase difference values $\Delta \phi_{j}(x,z)=\phi_{j}(x,z)-\phi_{ref}(x,z)$ prior to phase balancing and complex averaging. For this purpose, the phase differences $\Delta \phi_{j}(x,z)$ are first calculated between the *j*-th frame and the reference frame. In order to minimize the number of phase wraps in presence of strong axial motion differences, we choose the central frame as a reference. Second, the *j*-th phase difference B-scan is averaged using the respective phasors within a small kernel which may, for instance, be rectangular:

(42) $$\bar{\Delta \phi_{j}}(x,z)=\text{arg}\;\left[\sum_{x=x-\Delta x/2}^{x+\Delta x/2}\sum_{z=z-\Delta z/2}^{z+\Delta z/2}A_{j}(x,z)A_{ref}(x,z)\exp\left[i\Delta \phi_{j}(x,z\right)]\right].$$

Ideally, the kernel size (Δx,Δz) is chosen large enough to include at least a few speckles but small enough to only cover a patch of similar axial motion. The PDF of the averaged phase difference $\bar{\Delta \phi_{j}}(x,z)$ centered at zero can be derived from Eq. (2) by performing a variable transform to polar coordinates and then integrating the PDF over the amplitude space [47], whereby $\sigma_{\Delta \phi }=\sqrt{2}\sigma$ is used for the standard deviation:

(43) $$\begin{array}{rl} p_{\Delta \phi }(\Delta \phi ,A)= & \frac{1}{2\pi }\exp\;\left[-\frac{A^{2}}{4\sigma_{\Delta \phi }^{2}}\right]+\\ & +\frac{A}{\sqrt{4\pi }\sigma_{\Delta \phi }}\cos\Delta \phi \exp\;\left[-\frac{A^{2}\sin^{2}\Delta \phi }{4\sigma_{\Delta \phi }^{2}}\right]\;\text{erf}\;\left[\frac{A}{\sqrt{2}\sigma_{\Delta \phi }}\cos\Delta \phi \right] \end{array}$$

where $\text{erf}(u)=\frac{1}{\sqrt{2\pi }}\int_{-\infty }^{u}\exp\;\left[-v^{2}/2\right]dv$ denotes the Gaussian error function. With increasing $A/\sigma_{\Delta \phi }$, the distribution $p_{\Delta \phi }$ becomes more narrow and converges towards a δ-function at Δϕ=0 [22,47]. For Δϕ≠0, the maximum of the distribution is located at the respective expectancy value for the phase difference. In absence of a signal A, the somewhat complicated PDF $p_{\Delta \phi }$ reduces drastically and becomes a uniform distribution on (−π,π),

(44) $$p_{\Delta \phi }(\Delta \phi ,A=0)=\frac{1}{2\pi }.$$

Hence, the averaging process in Eq. (42) provides an estimate of the localized phase shift Δϕ with improved precision in case signal pixels are included within the averaging kernel (Δx,Δz) but delivers a random $\bar{\Delta \phi }$ if the kernel only includes noise.

If now, in a third step, the locally averaged phase difference map (Eq. (42)) is used to correct the phasors of the *j*-th frame with respect to the reference frame,

(45) $$S_{OCT}^{\left(j,\bar{moco}\right)}(x,z)=\sqrt{A_{j}(x,z)A_{ref}(x,z)}\exp\;\left[i\;(\phi_{j}(x,z)-\bar{\Delta \phi_{j}}(x,z))\right],$$

the phasors will only be aligned for signals but will retain their random character for noise regions. By averaging N such axial motion balanced frames, the full potential of complex averaging can be tapped without suffering from signal reduction due to local displacement differences.
